# Integrating Knowledge-Based and Machine Learning for Betel Palm Mapping on Hainan Island Using Sentinel-1/2 and Google Earth Engine

**DOI:** 10.3390/plants14172696

**Published:** 2025-08-28

**Authors:** Hongxia Luo, Shengpei Dai, Yingying Hu, Qian Zheng, Xuan Yu, Bangqian Chen, Yuping Li, Chunxiao Wang, Hailiang Li

**Affiliations:** 1 Institute of Scientific and Technical Information, Chinese Academy of Tropical Agricultural Sciences/Key Laboratory of Applied Research on Tropical Crop Information Technology of Hainan Province, Haikou 571101, China; xxs_lhx123@catas.cn (H.L.);; 2 Hainan Tang Huajun Academician Workstation, Haikou 571101, China; 3 College of Geography and Environmental Science, Hainan Normal University, Haikou 571158, China; 4 Hainan Danzhou Agro-Ecosystem National Observation and Research Station, State Key Laboratory Incubation Base for Cultivation & Physiology of Tropical Crops, Rubber Research Institute (RRI), Chinese Academy of Tropical Agricultural Sciences (CATAS), Haikou 571101, China; 5 Hainan Geomatics Centre, Ministry of Natural Resources, Haikou 570203, China

**Keywords:** betel palms (*Areca catechu* L.), logistic regression, machine learning algorithm, Sentinel-1/2 images, Google Earth Engine

## Abstract

The betel palm is a critical economic crop on Hainan Island. Accurate and timely maps of betel palms are fundamental for the industry’s management and ecological environment evaluation. To date, mapping the spatial distribution of betel palms across a large regional scale remains a significant challenge. In this study, we propose an integrated framework that combines knowledge-based and machine learning approaches to produce a map of betel palms at 10 m spatial resolution based on Sentinel-1/2 data and Google Earth Engine (GEE) for 2023 on Hainan Island, which accounts for 95% of betel nut acreage in China. The forest map was initially delineated based on signature information and the Green Normalized Difference Vegetation Index (GNDVI) acquired from Sentinel-1 and Sentinel-2 data, respectively. Subsequently, patches of betel palms were extracted from the forest map using a random forest classifier and feature selection method via logistic regression (LR). The resultant 10 m betel palm map achieved user’s, producer’s, and overall accuracy of 86.89%, 88.81%, and 97.51%, respectively. According to the betel palm map in 2023, the total planted area was 189,805 hectares (ha), exhibiting high consistency with statistical data (R^2^ = 0.74). The spatial distribution was primarily concentrated in eastern Hainan, reflecting favorable climatic and topographic conditions. The results demonstrate the significant potential of Sentinel-1/2 data for identifying betel palms in complex tropical regions characterized by diverse land cover types, fragmented cultivated land, and frequent cloud and rain interference. This study provides a reference framework for mapping tropical crops, and the findings are crucial for tropical agricultural management and optimization.

## 1. Introduction

Betel palm (*Areca catechu* L.) is a critical commercial plantation crop, mainly distributed in Southeast Asia, South Asia, the Pacific, and parts of Africa [1], and holds significant importance for the tropical regions of China [2]. Hainan Island is the main producing region of betel palms, accounting for 95% of China’s betel nut acreage [3]. The planting regions of betel palms on Hainan Island increased from 4.77 × 10^4^ ha in 2005 to 1.82 × 10^5^ ha in 2022, and the corresponding production of betel nuts rose from 6.43 × 10^4^ tons in 2005 to 2.92 × 10^5^ tons in 2022. The direct gross annual value of betel nuts was USD 4.92 × 10^9^ in 2019, contributing 7.1% to Hainan’s Gross Domestic Product (GDP) [4]. Thus, the betel palm industry is extremely important for tropical rural revitalization. Due to the considerable economic benefits, rapid expansion of betel palms by converting natural forests and establishing stands has been observed [5]; however, this conversion has led to various eco-environmental problems, including species richness reduction, soil erosion, and decreased carbon sink [1,6]. Additionally, the controversy surrounding betel nut consumption, linked to oral carcinoma [7], has encouraged more and more departments to impose bans on the sale of betel nut products [8], significantly impacting the industry. Therefore, accurate and timely mapping of betel palm plantations is vital for effective environmental monitoring and guiding the sustainable development of betel palm cultivation [9].

The rapid advancement of remote sensing technology provides an effective and convenient means for accurate and objective vegetation classification across regional and global scales [10]. Currently, the publicly accessible medium-resolution spatial data has been extensively utilized in identifying vegetation across large region scales [11]. Long-term series of Sentinel-2 images feature a 5-day revisit cycle, providing a new opportunity for large-scale vegetation mapping. Compared with other medium-resolution optical images, Sentinel-2 images have a broader range of spectral bands, significantly enhancing their potential for detailed vegetation analysis. In particular, the three red edge bands, located at wavelengths of 705, 740, and 783 nm, respectively, provide more supplemental information for precision vegetation classification, as they cover very narrow parts of the spectrum that traditionally only appear in hyperspectral sensors [12]. Vegetation indices acquired from spectral bands enhance the capacity of identifying essential information for vegetation types. Therefore, Sentinel-2 images have become more and more popular in current land cover classification methods [13]. However, in tropical regions, the land surface is often obscured by the constant presence of rain or cloud [14], leading to more challenges for crop mapping based on optical images over a large scale. Synthetic aperture radar (SAR) data, as an active system, which is not affected by cloudy or rainy coverage, offers an alternative to optical data for distinguishing land cover types [15]. Due to the difference between SAR and optical sensors in interaction with vegetation, data derived from the SAR system supply abundant complementary information (mainly polarization components) for mapping vegetation types [16]. Recently, an increasing number of research efforts have integrated optical and SAR images for identifying land cover types, including plantations [17,18], crops [16,19], and even single tree species [20]. Overall, many scholars have highlighted that the integration of optical and SAR images can significantly improve classification accuracy.

In terms of mapping tropical vegetation at large scales, previous efforts have mainly focused on rubber plantations [21], oil palms [22], eucalyptus [23], and others. Fewer studies have used optical and SAR images for betel palm mapping. Rubber plantations have a distinct defoliation phase, while eucalyptus plantations have a short rotation of 5–6 years. Thus, both present unique vegetation variations in time series remote sensing data. Industrial oil palm plantations can be identified by their unique dense road networks [17]. Indeed, more challenges will appear in betel palm mapping compared to the aforementioned tropical crops. As a perennial and evergreen crop, betel palms lack distinct phenological features, making them more challenging to identify. Furthermore, due to rapid population growth, the average farm size in tropical regions is less than 1 ha, leading to increased fragmentation of agricultural landscapes, with smallholders dominating much of tropical production. The fragmentation and diversification of tropical regions have reduced spectral separability between betel palms and other tropical vegetation [24], further complicating their identification in a complex tropical landscape. Therefore, it is necessary to develop an appropriate method to accurately distinguish betel palms from other complex crop types at large regional scales.

With the advancement of machine learning (ML) techniques, ML algorithms, such as Random Forest (RF) [25], Support Vector Machines (SVM) [26], Gradient Boosting Decision Trees (GBDT) [27], and deep learning models, have demonstrated strong capabilities in handling the high-dimensional, nonlinear relationships inherent in multi-source remote sensing data. These methods not only improve classification accuracy but also enhance model adaptability to diverse ecological and environmental conditions. In addition, knowledge-based approaches, unlike data-driven algorithms, can be directly applied across different regions and time periods without requiring retraining, making them particularly useful for large-scale applications [28]. The construction knowledge-based mapping approaches fundamentally rely on establishing the relationship between crop attributes and remote sensing variables, which requires extensive domain expertise [19]. Previous studies have revealed that knowledge-based methods can effectively capture the difference among vegetation types [29]. Consequently, the integration of knowledge-driven and machine learning techniques has emerged as a promising direction for enhancing land cover classification and crop mapping, especially in regions with complex land cover patterns.

When using traditional remote sensing methods, handling large-scale datasets becomes a major challenge, particularly on workstation-based systems reliant on commercial software [30]. In recent years, however, rapid advances in computational power have addressed these limitations. Cloud platforms such as Google Earth Engine (GEE) leverage extensive global server networks and cutting-edge cloud computing technologies to store and process massive volumes of Earth observation data [31]. By providing access to an extensive archive of satellite imagery, GEE enables researchers to efficiently analyze trillions of images in a parallelized framework, thereby revolutionizing large-scale environmental and geospatial research. Numerous recent studies had utilized the GEE platform for large-scale land cover mapping [32,33,34].

Based on the above-mentioned background, this study pursues two main objectives: (1) to design an effective framework that integrates knowledge-based and machine learning algorithms on the GEE for betel palm identification, and (2) to assess the potential of combining optical (Sentinel-2) and SAR (Sentinel-1) data to distinguish betel palm patches within complex tropical agricultural landscapes, which is important for optimizing the planting structure of betel palms.

## 2. Results

### 2.1. Comparison of Different Forest Maps

**Figure 1:**
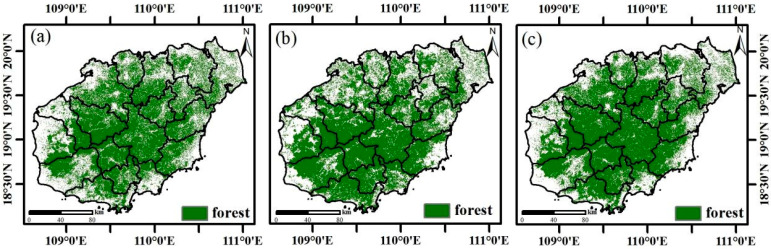
Different forest resultant maps. (**a**) Forest map in this study, (**b**) GlobalLand 30 forest map (2020), (**c**) CLCD forest map (2022).

Forest area estimates for Hainan Island derived from Sentinel-1/2, GlobalLand30 and China Land Cover Dataset (CLCD) maps were 2.18 × 10^6^ ha, 2.20 × 10^6^ ha and 2.21 × 10^6^ ha, respectively. According to the Hainan Statistical Yearbook [35], the officially reported forest area in 2023 was 2.14 × 10^6^ ha, closely aligning with Sentinel-1/2 estimates. Despite differences in satellite sensors and classification methods, all three maps exhibited similar spatial patterns of forest distribution across the island (Figure 1).

**Figure 2:**
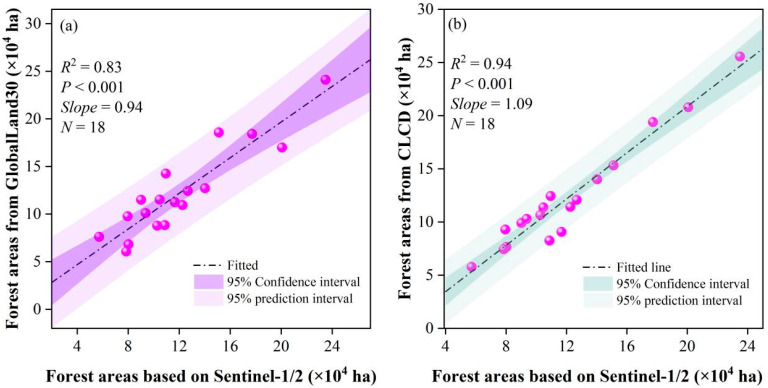
Scatter plots and fitted lines comparing forest areas between the Sentinel-1/2 map and other forest datasets. (**a**) Sentinel-1/2 and GloblalLand30, (**b**)Sentinel-1/2 and CLCD.

At the county scales, significant linear correlations were observed among the three forest maps (Figure 2). The forest maps derived from Sentinel-1 demonstrated strong relationships with those from GlobalLand30 (R^2^ = 0.83, *p* < 0.001) and CLCD (R^2^ = 0.94, *p* < 0.001), respectively. The regression slopes (0.94 and 1.09, respectively) further indicate a high degree of proportional consistency in forest area estimations. These findings suggest that the Sentinel-1/2-based forest map is not only consistent with existing national land cover products but also provides higher spatial resolution (10 m), making it a reliable baseline for further extracting betel palm plantations in complex tropical landscapes.

### 2.2. Delineating Key Features for Mapping Betel Palms

**Figure 3:**
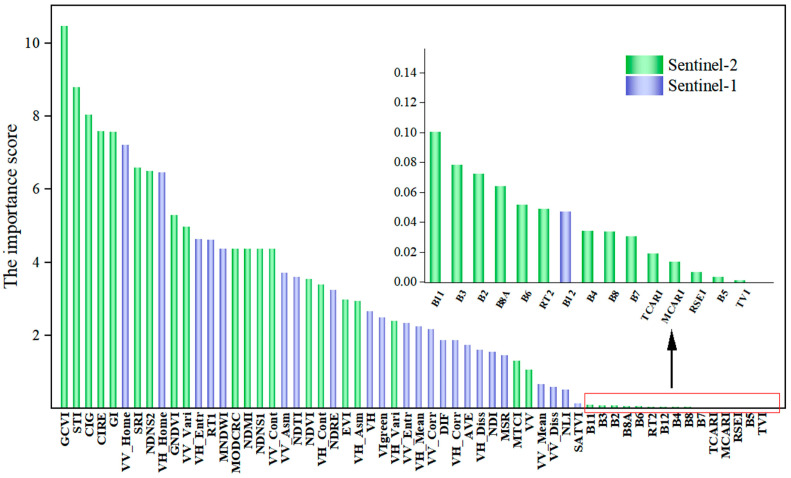
Feature importance of variables based on logistic regression (LR).

The detailed results of feature importance analysis are shown in Figure 3. Among all variables, Green Chlorophyll Vegetation Index (GCVI) emerged as the most important variable for betel palm classification, with an importance score of 10.46. Simple Tillage Index (STI) and Chlorophyll Index Green (CIG) ranked second and third, with scores of 8.81 and 8.03, respectively. These results emphasize the critical role of Short-Wave Infrared (SWIR) and red-edge bands for capturing vegetation traits and disturbance signals. Among the top ten variables, eight were vegetation indices and two were SAR-derived textural features. Furthermore, the classification accuracy reached a plateau once the number of input features exceeded 20, suggesting limited performance improvement beyond this threshold. For comprehensive consideration of classification precision and computational efficiency in large-scale mapping, the top 20 features were selected for betel palm identification, comprising GCVI, STI, CIG, Chlorophyll Index Red Edge (CIRE), Greenness Index (GI), VV-Home, Simple Ratio Index (SRI), Normalized Difference NIR and SWIR2 Index (NDNS2), VH_Home, GNDVI, VV_Vari, VH_Entr, RT1, Modified Normalized Difference Water Index (MNDW), Modified Crop Residue Cover (MODCRC), Normalized Difference Moisture Index (NDMI), Normalized Difference NIR and SWIR1 Index (NDNS1), VV_Cont, VV_Asm and VH. Specifically, optical features accounted for 70% of the selected predictors, while SAR-based features contributed 30%, further highlighting the synergistic benefits of integrating multi-source data in betel palm mapping.

### 2.3. Accuracy Assessment and Geographical Characteristics of Betel Palms

Accuracy assessment is an indispensable step in remote sensing map production. The classification performance for betel palms was evaluated using testing samples, including betel palm samples (1763 pixels) and non-betel palm samples (15,273 pixels). The classification confusion matrix, including producer’s, user’s, overall accuracy and Kappa coefficient, is shown in Table 1. Results showed a producer’s accuracy of 88.81% (11.19% omission error) and a user’s accuracy of 86.89% (14.11% commission error), indicating both high and well-balanced accuracy levels. Overall accuracy reached 97.51%, with a corresponding kappa coefficient of 0.86, demonstrating the method’s strong reliability for identifying betel palms from other land cover types on Hainan Island.

Field surveys and high-resolution Google Earth imagery further revealed that misclassifications were predominantly concentrated in dense mountain forests and artificial plantations, particularly coconut palms.

**Figure 4:**
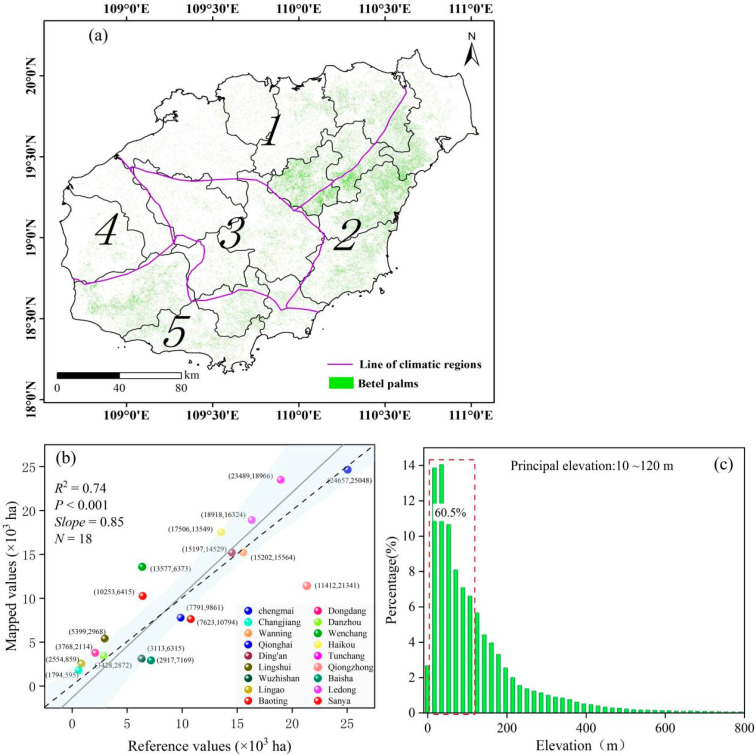
Mapping results and spatial and topographical factors of betel palm distribution on Hainan Island. (**a**) Distribution map of betel palms across Hainan Island. Climatic zones: 1, semi-humid zone; 2, humid zone; 3, mountainous humid zone; 4, semi-arid zone; and 5, semi-arid and semi-humid zone. (**b**) Scatter plot of betel palm areas comparing statistical data and mapping results at the county scale. (**c**) Histogram showing the proportion of betel palm distribution across different various elevation ranges.

Figure 4a presents the 10 m resolution classification map of betel palms on Hainan Island in 2023. Each 10 m pixel represents 0.01 hectares (ha), enabling not only analysis at the large scale but also at the level of individual farms, regardless of their size. The total estimated area of betel palms was 189,805 ha, slightly overestimating the official 2023 value of 181,656 ha by 4.49%. Country-level analysis (Figure 4b) revealed that betel palms were concentrated in Qionghai, Tunchang, Ledong, Haikou, Ding’an and Wan’ning, with their location mainly concentrated in these regions where climatic and topographic conditions favor betel palm cultivation. These six counties accounted for approximately 1.15 × 10^5^ ha, or 60.65% of the total area. Upon comparing the classified results with statistical data from 18 counties, a high consistency was observed, with an R^2^ of 0.74 and a slope of 0.85. Notably, overestimation was most evident in Tunchang and Haikou, whereas underestimation occurred primarily in Baisha (Figure 4b).

Betel palm distribution is shaped by both environmental factors, such as climate and topography, and economic drivers, notably profitability. Betel palms grow optimally in regions with high annual precipitation and relatively uniform rainfall throughout the year, making them particularly suited to humid tropical environments. Due to their limited drought tolerance, betel palm cultivation is typically restricted to areas with stable and abundant moisture availability [36].

Although the overall distribution pattern of betel palms across Hainan Island appears relatively scattered, visual inspection revealed that they are mainly concentrated in the eastern, central, and southwestern regions (Figure 4a). These areas were characterized by a warm and humid climate that provided favorable conditions for betel palm cultivation. Among them, the eastern region, featuring flat terrain, fertile soil, and well-developed agricultural infrastructure, had become a major production zone. Furthermore, topographic analysis from the resultant 10 m resolution map (Figure 4c) further showed that betel palms were primarily distributed in low-altitude regions, with most occurring between 10 and 120 m.

**Figure 5:**
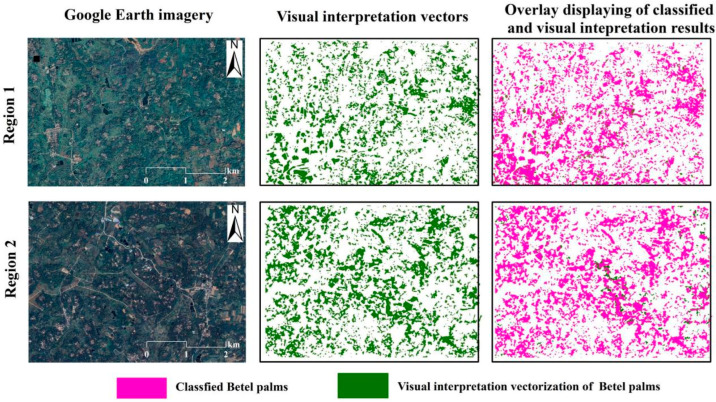
Comparison between the betel palm map and the visual interpretation of high-resolution imagery. Note: Region 1 from Qionghai City, Hainan Province (110°33′21″ E, 19°24′58″ N), and Regions 2 from Wanning City, Hainan Province (110°23′52″ E, 18°56′39″ N).

To further evaluate classification accuracy, two representative regions were selected for detailed validation. Within these regions, the boundary vectors of betel palm plantations were delineated from high-resolution Google Earth imagery and supplemented with field surveys. Figure 5 compares the classification results with the manually interpreted plantation boundaries. The overlay maps demonstrated a high degree of spatial consistency between the classified outputs and the visually labeled reference data, indicating that the classification model effectively captured actual betel palm distribution patterns.

## 3. Discussion

### 3.1. Mapping Algorithm for Forest

This study proposes a novel framework for mapping betel palms on Hainan Island using Sentinel-1/2 imagery integrated with a knowledge-driven machine learning classifier. For large-scale vegetation classification, the knowledge-based approach effectively mitigated of intra-class variability and inter-class similarity [28], emphasizing the relationships between remote sensing features and vegetation attributes [19].

SAR backscatter attributes were first analyzed across land cover types, and their distinct signatures were used to delineate forests on Hainan Island. Subsequently, non-forest regions were excluded before betel palm mapping, substantially reducing computational requirements compared with conventional supervised classification [29]. Sentienl-1 has been a popular data resource in forest identification [37,38], owing to its fine spatial resolution and frequent revisit capability. Forests exhibited higher VV and VH values than water, consistent with previous study [16]. Nonetheless, notable overlap was observed between forest and cropland, both in raw backscattering attributes (VV and VH) and in derived indices (average (AVE), difference (DIF), Ratio1 [RAT1], Ratio2 [RAT2], Normalized Difference Index (NDI), and NL Index (NL)), reflecting the island’s complex cropping structures. 

The GNDVI derived from Sentinel-2 effectively reduced forest misclassification at a threshold of 0.69. The GNDVI is more sensitive to chlorophyll variation than Normalized Difference Vegetation Index (NDVI), which is commonly employed to evaluate canopy biomass changes [39]. As a result, forest exhibited higher GNDVI values than cropland in this study. The forest map generated from the combined Sentinel-1/2 data showed good consistency with two existing forest products (GlobalLand30 and CLCD) on Hainan Island. In contrast, there were higher correlations between Sentinel-1/2 and CLCD forest maps (R^2^ = 0.94). Spatial discrepancies among the three forest maps can be attributed to differences in production time, data sources, and classification algorithms. For example, the production year of Sentinel-1/2 (2023) forest was closer to that of CLCD (2022) compared to GlobalLand30 (2020). In addition, the annual CLCD was derived from a post-processing approach integrating spatial–temporal and logical reasoning [40], whereas Globalland30 was generated from the incorporation of pixel-based and object-oriented classification approaches with knowledge-guided interaction [41].

### 3.2. The Contribution of Optimal Variable Selection for Mapping Betel Palms

This study analyzed remote sensing features associated with betel palm crop, and subsequently extracted optimal features for identifying betel palms across Hainan Island. Previous studies have shown that feature optimization can significantly increase classification precision of land cover mapping [42,43]. Unlike forests, which exhibit apparent phenological features, selecting optimal variables for mapping evergreen betel palms is both more challenging and critical. For example, rubber plantations undergo pronounced winter defoliation lasting nearly 2 months [44]; therefore, the dominant features can be easily determined even without feature selection algorithms. In contrast, there is an absence of prominent phenological traits in betel palms, which highlights the necessity of identifying optimal variables to achieve accurate classification. In this study, LR was employed to identify key variables, as it has demonstrated strong performance when integrated with an RF classifier for tropical vegetation mapping [25].

The analysis results identified GCTV, STI, CIG and CIRE as the four most important variables for betel palm identification. GCTV and CIG are chlorophyll-related indices that directly reflect vegetation photosynthetic capacity, developmental stages and canopy stress [45]. Several studies have emphasized the physical differences in chlorophyll content among diverse crop types [3,46]. STI, derived from SWIR1 and SWIR2 channels, effectively captures non-pigment biochemical properties of vegetation [19,47], underscoring the importance of SWIR bands for betel palm mapping. Compared to other forests, betel palm growth demands sufficient water [48], making canopy moisture content easier to capture in SWIR bands. Many studies have noted the excellent performance of SWIR1 and SWIR2 in recognizing complex crops, such as tea, maize, soybean, rice [49,50]. The high ranking of CIRE is also justified, as it is derived from red edge bands, known for their sensitivity to vegetation stress and forest changes [51].

SAR backscatter is mainly influenced by canopy permittivity and canopy morphology [19], with VV and VH polarizations associated with the top of tree crown and tree crown structure, respectively [52]. This highlights the potential of SAR data for canopy characterization. When analyzing features derived from Sentinel-1 SAR images, additional texture information, such as homogeneity, significantly contributed to good performance in betel palm classification. Su et al. [53] also demonstrated that Gray-Level Co-occurrence Matrix (GLCM) homogeneity was highly effective in mapping forests and dense crop regions. The pinnately compound leaves of the betel palm, with long linear blades (30–60 cm length and 2.5–4 cm width), form a smooth green crown shaft that produces a distinctive canopy structure. This morphology yields strong backscatter in both polarizations, creating a characteristic homogeneity that differentiates betel palms from other tropical forests on Hainan Island (Figure 3).

### 3.3. The Advantages of Incorporating Optical and SAR Data for Mapping Betel Palms

Utilizing Sentinel-1 and Sentinel-2 data, betel palm classification on Hainan Island achieved an overall accuracy of 97.51%, with user’s and producer’s accuracies of 86.89% and 88.81%, respectively. These results demonstrate the effectiveness of integrating optical and SAR data for identifying betel palms in complex tropical forests. The optical images of Sentinel-2 provide a wealth of band information for identifying complex vegetation types. Different tropical forests have distinct characteristics of Leaf Area Index (LAI), which is a critical indicator representing the growth and canopy architecture of forests [54]. Generally, tropical rainforest exhibits higher LAI compared to artificial forests, making it easier to detect canopy change in different tropical forests. For example, the mean LAI of mature rubber plantation and betel palm were around 3 m^2^/m^2^ and 1.6 m^2^/m^2^, respectively, while that of natural forest showed a significantly higher value (>5 m^2^/m^2^) [55,56]. The high classification performance of betel palm mapping could be attributed not only to the spectral differences among tropical forest types, but also to the contribution of SAR polarization data. Previous studies have demonstrated that combining optical and radar data can significantly enhance classification accuracy in cloud-prone regions compared to other regions [57], as radar data is unaffected by could cover [21].

**Figure 6:**
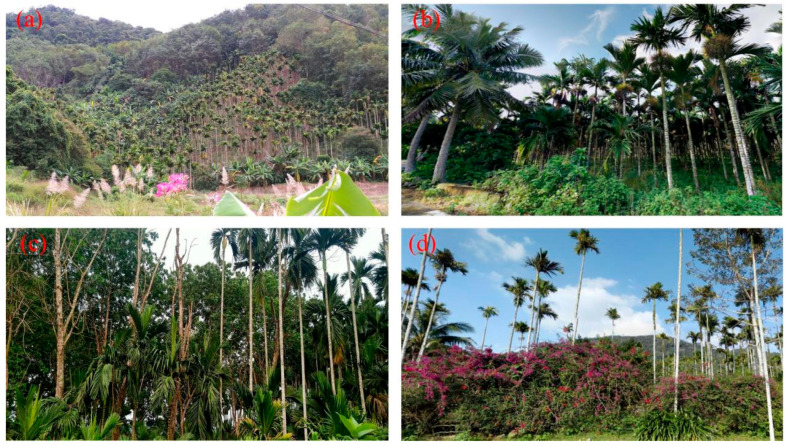
Different planting conditions of betel palms and other land cover types. (**a**) Betel palms replacing the tropical forest (109°25′23″ E, 18°31′22″ N), (**b**) betel palms planted beside coconut palms (110°31′04″ E, 19°25′10″ N), (**c**) intercropping of betel palms and rubber plantations (110°6′33″ E, 19°31′15″ N), and (**d**) sparse betel palms, intermixed with Bougainvillea spectabilis (109°10′51″ E, 18°27′43″ N).

Visual inspection of the classification map against high-resolution remote images revealed misclassifications between betel palms and other forests, particularly coconut palms and sparse tropical rainforest, even after applying LR-based feature selection. Similar issues have been reported in global coconut palm mapping [58]. The fragmented land-use pattern on Hainan Island, largely shaped by smallholder management [59], further complicates classification. Unlike industrial plantations, many betel palms are cultivated adjacent to coconut palms or tropical forest (Figure 6a,b). Field surveys also indicated that some farmers are cultivating betel palms near rubber plantations due to the declining profitability of rubber plantations (Figure 6c), while others cultivate them sparsely in mixed stands with shrubs or vines (Figure 6d). Such planting practices create spectral and textural similarities among land cover types, contributing to the observed misclassification.

### 3.4. Uncertainties and Future Directions

Although the classification achieved high accuracy, certain limitations remain in mapping betel palms. Pixel-based approaches inevitably produce mixed pixels, particularly in transitional areas between young betel palms and surrounding forests. Field surveys confirmed that some young betel palms were intercropped with rubber plantations or other perennial crops, making their detection difficult. According to the Hainan Statistical Yearbook [35], newly planted betel palms covered 7.74 × 10^3^ ha in 2022, yet these areas were not reliably identified in this study, contributing to discrepancies between our results and official statistics (Figure 4b). Future work should prioritize the detection of young betel palms to better support the sustainable industry development on Hainan Island. Additionally, state-of-the art classification algorithms, such as deep learning, ought to be introduced in complex tropical classification.

## 4. Materials and Methods

### 4.1. Study Area

**Figure 7:**
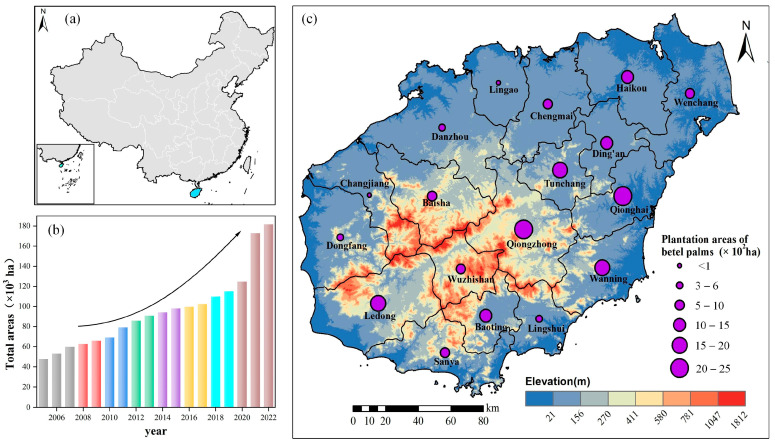
General overview of Hainan Island. (**a**) Geographical location of Hainan Island in China, (**b**) total planting areas of betel palms on Hainan Island from 2005 to 2022, and (**c**) topography of Hainan Island and betel palm planting areas across counties or cities.

Hainan, China’s second-largest tropical island, covers 33,900 km^2^ and has an elliptical terrain with a hilly core surrounded by flat coastal plains (Figure 7). The island experiences a typical tropical and subtropical monsoon climate, with average annual temperatures of 22–27 °C and mean annual precipitation of 1600–1700 mm. Additionally, Hainan Island shows the significant characteristic of wet (from May to October) and dry (from November to April) seasons.

In 2022, forests covered over 62.1% Hainan Island, comprising both natural and artificial forests. Due to the demands of economic development, many natural forests have been transformed into planted forests [6,21]. Among these, rubber plantations, betel palms and coconut palms are regarded as the island’s three primary economic forests by the local government.

### 4.2. Datasets and Processing

#### 4.2.1. Satellite Data

To map betel palms in Hainan Island, both optical images (Sentinel-2) and SAR images (Sentinel-1), provided by the European Space Agency, were used in this study. All image pre-processing was implemented in Google Earth Engine (GEE).

The Sentinel-1 dataset primarily consists of two backscatter bands in the C-band: dual cross-polarization (VH: vertical transmit/horizontal receive) and single co-polarization (VV: vertical transmit/vertical receive) bands [17]. In this study, Sentinel-1 images were sourced from Ground Range Data (GRD) data with the Interferometric Wide Swath (IW) mode, with a spatial resolution of 10 m [57]. Pre-processing steps involved orbital file correction, removal of GRD border and thermal noise, radiometric calibration, and terrain correction [60]. Subsequently, to eliminate speckle-noise, the Refine Lee filter was applied.

Meanwhile, the Sentinel-2 dataset of level-2A was used, comprising 12 spectral bands: four bands at 10 m resolution, six bands at 20 m resolution, and three bands at 60 m resolution, scaled by 10,000. All bands were resampled to 10 m for consistency in the analysis. Then, these products were ortho-rectified and UTM-geocoded to Top-of-Atmosphere reflectance using sub-pixel multispectral and multi-data registration. To ensure high image quality, the threshold of cloud cover was set to less than 10%. An automated cloud masking algorithm was used to remove opaque and cirrus clouds based on the bitmask band of QA60. Ultimately, a total of 1480 images were selected for mapping betel palms. Given the frequent cloud cover and rainfall in tropical regions, a median composite method was applied, combining the Sentinel-2 data pixel by pixel across the entire study region.

#### 4.2.2. Forest Dataset

To evaluate the forest classification results on Hainan Island, two 30 m resolution datasets were employed: GlobalLand30 (https://shop.geospatial.com/publication/XV1CCGGP7TGJ44PGKT0RJ15V47/GlobeLand30-30-meter-Global-Land-Cover (accessed on 13 July 2025)) and China Land Cover Dataset (CLCD) (https://doi.org/10.5281/zenodo.4417810). GlobalLand30 was acquired from the National Geomatics Center of China (NGCC), and was primarily classified based on Landsat data and Chinese Environmental and Disaster satellite images [41]. The dataset currently includes three periods (2000, 2010, and 2020) of global land cover images. The CLCD dataset, released by scholars at Wuhan University, contained annual land cover images from 1985 to 2022 in China [40]. In particular, it was produced by Landsat OLI/ETM+/TM imaging, providing long-term serial information of land cover and land use for global change research. For this paper, the 2020 GlobalLand30 and 2022 CLCD datasets were selected, as they align most closely with the 2023 study period. Both GlobalLand30 and CLCD maps were clipped to the extent of Hainan Island using a vector map of the island.

#### 4.2.3. Sample Data

Ground reference data were crucial for identifying betel palms; these data were obtained from field surveys and Very High Resolution (VHR) imagery on Google Earth. Field surveys were conducted in December 2023 using handheld Global Positing System (GPS) receivers and digital cameras. Six land cover categories were defined: betel palms, natural forest, rubber plantations, croplands (e.g., vegetables, banana plantations, mango plantations), built-up areas (roads and buildings), and water. Based on field survey data and VHR images from Google Earth in 2023, we finally selected 2225 regions of interest (ROIs) across six land cover types (Table 2), using a stratified random sample. The ROIs were randomly split into testing and training data at a ratio of 3:7. In the GEE code editor, training samples were imported via the JavaScript API through Google fusion tables.

### 4.3. Mapping Algorithms

**Figure 8:**
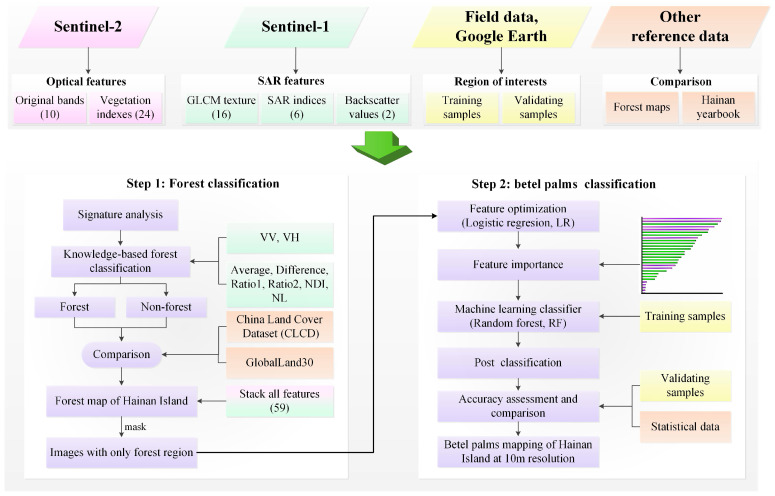
Workflow for mapping betel palms of Hainan Island based on Sentinel-1 and Sentinel-2 imagery.

The detailed workflow for mapping betel palms is outlined in Figure 8. First, the Sentinel-1 and Sentinel-2 data were pre-processed in GEE, forming a stacked dataset with 58 features. Second, after performing a signature analysis of Sentinel-1, a preliminary forest map of Hainan Island was generated using a knowledge-based approach. Then, the GNDVI calculated from Sentinel-2 was used to remove misclassified cropland pixels from the forest map. Third, the stacked datasets were subsequently masked with the forest map, leaving only forest areas. LR was applied for feature optimization to identify the optimal features for mapping betel palms. Finally, betel palms were distinguished from other forests using a random forest classifier and the optimized features.

#### 4.3.1. Feature Selection from Sentinel-1

Given the structural characteristics of betel palms, eight texture features derived from Gray level Co-occurence Matrix (GLCM) were calculated as an average of over four spatial orientations (0°, 45°, 90°, 135°). Detailed information of GLCM texture metrics are shown in Table 3. To explore the optimal moving window size, we compared window sizes of 3 × 3, 5 × 5, 7 × 7, 9 × 9, 11 × 11 pixels in this study. Ultimately, we found that 5 × 5 pixel moving windows effectively capture fine details, particularly for distinguishing between natural forest and planted forests (primarily rubber plantations and betel palms). Previous studies have also demonstrated the effectiveness of Sentinel-1-derived texture features for tropical agricultural landscapes [18].

To enhance the detailed information for mapping betel palms, six indices from Sentinel-1 were all computed in GEE and combined into a stack, including average (AVE), difference (DIF), Normalized Difference Index (NDI), NL Index, and simple ratios of VV and VH (RAT1 and RAT2). These indices have demonstrated good performance in identifying oil palms [22], coconut palms [61], rubber plantations and tropical forests [62].

#### 4.3.2. Feature Selection from Sentinel-2

For Sentinel-2 images, 34 features were used to identify betel palms (Table 4), through referring to relevant literature. Spectral indices, which were sensitive to vegetation greenness and water condition, showed a tremendous potential for capturing physical discrepancies among land cover types [11]. In addition to the 10 spectral bands, 24 vegetation indices were derived to distinguish the characteristics of different forests (Table 4).

#### 4.3.3. Feature Optimization

To reduce feature redundancy and enhance classification stability, variable importance was assessed using LR. Additionally, our previous study found that the combination of LR and machine learning algorithms (such as Random Forest) could produce higher classification accuracy in tropical regions [25]. LR is a log-linear model commonly used in feature optimization [63], primarily describing the relationship between dependent and explanatory features based on a fitting regression model. The LR formula is expressed as follows:

(1) $$P(y_{i}=J)=\frac{1}{1-exp(\beta_{0i}+\beta_{1i}x_{1}+\beta_{2i}ix_{2}+\cdots +\beta_{ni}ix_{n})}$$

where J means the baseline category; $P(y_{i}=J)$ denotes the probability that the outcome $y_{i}$ falls into J; ($x_{1}$,$x_{2}$, ⋯, $x_{n}$) refer to the input variables; $\beta_{0i}$ is an intercept; and $\beta_{1i}$, $\beta_{2i}$, ⋯, $\beta_{ni}$ are the regression coefficients corresponding to each variable.

#### 4.3.4. Knowledge-Based Forest Mapping

**Figure 9:**
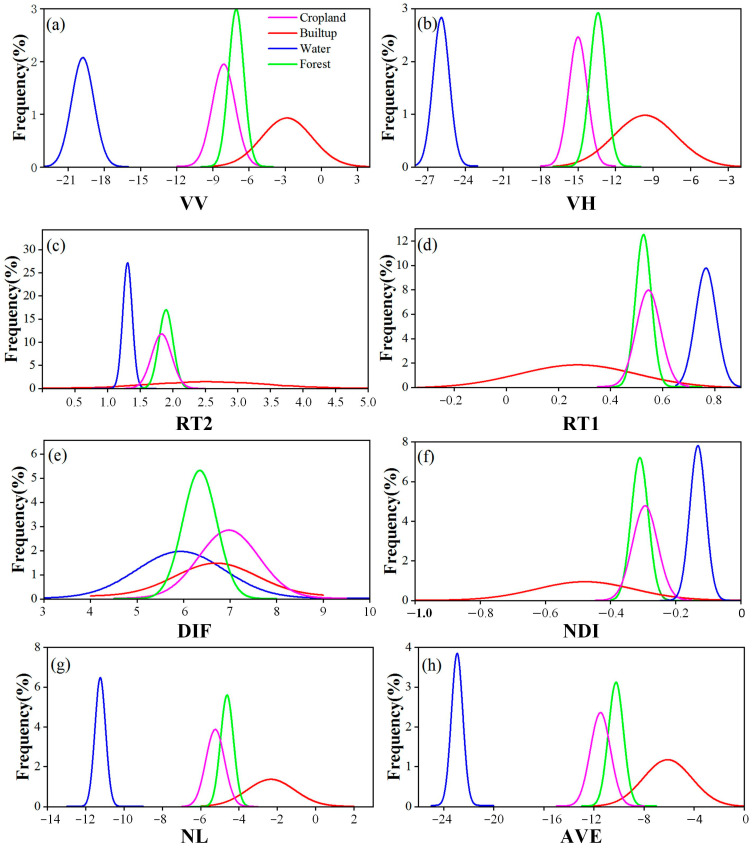
Backscatter signature of four land cover types across different polarization combinations. (**a**) VV, (**b**)VH, (**c**)RT2, (**d**) RT1, (**e**)DIF, (**f**) NDI, (**g**) NL, (**h**) AVE.

According to the classification criteria established by the Food and Agriculture Organization of the United Nations [64], land can be defined as forest if it spans over 0.5 hectares (ha), possesses a tree canopy cover larger than 10%, and includes trees capable of reaching more than 5 m in height. This definition has been applied in forest mapping studies [21,65]. A detailed workflow was developed for identifying forest on Hainan Island (Figure 2). Due to the good penetrating power of SAR, the interaction between incident energy and larger branch components of forest can produce substantial volume scattering [66], allowing SAR data to capture detailed forest characteristics, as demonstrated in prior literature [67]. In this study, the spectral signatures of four different land cover types (forest, cropland, built-up and water) were analyzed using training ROIs from Sentinel-1 satellite imagery. Frequency distributions were calculated from the backscatter values, containing two polarizations (VV and VH), Average (AVE), Difference (DIF), Ratio1, Ratio 2, Normalized Difference Index (NDI), NL Index (Figure 9).

**Figure 10:**
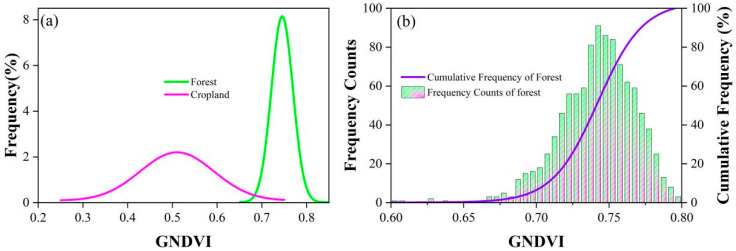
GNDVI histograms of cropland and forest. (**a**) Frequency histogram of GNDVI of cropland and forest. (**b**) Frequency counts and cumulative frequency of forest.

Water bodies, due to their smooth and calm nature, reflect most of the backscatter by mirror reflection [66]. Therefore, the histograms of VV, VH, RT2, NL and AVE for water showed even lower backscatter values than those of forest. The histograms of NDI and RT1 indicated that forest had a lower value compared to water. Both forests and built-up regions presented high VV and VH backscatter values, possibly attributable to the strong reflectance resulting from their intricate structural characteristics. Moreover, many built-up areas showed even higher values in VV and VH backscatters. NL and AVE effectively distinguished forests from built-up areas, while DIF overlapped with all other land cover types. Polarization-based features largely separated forests from water bodies, built-up areas, and most cropland, although some croplands exhibited backscatter patterns similar to forest (Figure 9). To further distinguish forest and cropland, GNDVI histograms of the two land types were analyzed (Figure 10). Misclassified forest pixels were removed using the 5th percentile threshold of GNDVI (0.69). Subsequently, a 5 × 5 median filter was used to eliminate the salt-and-pepper noise, resulting in the final forest map for Hainan Island.

### 4.4. Machine Learning Classifier

As an ensemble algorithm, random forest (RF) contains multiple tree-like classifiers [68]. Its computational efficiency makes it well suited for large-scale classification with massive datasets [56]. Unlike a single decision tree, RF exhibits excellent classification performance even with limited training data, and has proven to be robust against noise through bagging or boosting, thereby showing tremendous potential to handle large datasets. In GEE, five key hyperparameters are defined: numberOfTree, variablesPerSplit, minLeafPopulation, bagFraction, maxNodes, and seed. Based on the accuracy test, the numberOfTree was set to 100 for betel palm identification, aligning with previous research recommendations [55]. Apart from numberOfTree, all other parameters were set to the default values recommended by GEE. The default value of variablesPerSplit is set as the square root of the total number of features, a strategy widely supported in the literature and empirically validated as effective in mitigating overfitting [48]. The default bagFraction value is set to 0.5, indicating that 50% of the total samples are randomly selected through bootstrapping to construct each tree.

### 4.5. Post Classification

Pixel-based classification can efficiently identify land cover types over large areas but often produces salt-and-pepper noise compared with object-oriented approaches [69]. Post-classification processing improves the visual quality of initial mapping results by employing various methodologies. It was noteworthy that post-classification only improved the visual presentation, while the performance of mapping classifier was evaluated based on the classification map prior to post processing. To mitigate salt-and- pepper noise, a 5 × 5 median filter was used in the betel palm map through ENVI 5.3 software (Exelis Visual Information Solutions, Boulder, CO, USA).

### 4.6. Accuracy Evaluation

Accuracy evaluation is an efficient way to determine classification results, as it explores the alignment among the classified results and reference data, providing meaningful insights into misclassification patterns [70]. To quantitatively evaluate the classification performance, we employed standard accuracy metrics, namely overall accuracy (OA), kappa coefficient (kappa), user’s accuracy (UA), and producer’s accuracy (PA).

## 5. Conclusions

In this study, we proposed an integrated framework for mapping betel palms across Hainan Island using Sentinel-1/2 images within GEE, combining both knowledge-based and machine learning approaches. A high-precision 10 m resolution betel palm map was produced by first delineating forested areas using Sentinel-1 backscatter features and Sentinel-2-derived GNDVI, producing a forest mask with strong spatial agreement with the GlobalLand30 and CLCD datasets. Subsequently, betel palm plantations were identified from forest areas using an RF classifier in conjunction with feature selection based on LR. Feature importance analysis revealed that vegetation indices dominated the top-ranked features, with GCVI, STI, and CIG being the most critical, highlighting the importance of red-edge and SWIR bands in capturing betel palm-specific vegetation traits. Of the top 20 features, 70% were optical and 30% SAR-derived, demonstrating the complementary value of multisource data. Classification achieved a user’s accuracy of 86.89% (commission error 13.11%) and a producer’s accuracy of 88.81% (omission error 11.19%). The final 2023 betel palm map indicated a total plantation area of 189,805 ha, closely aligning with statistical yearbook data, with an R^2^ of 0.74. The spatial distribution was predominantly concentrated in the eastern part of the island, where climatic and topographic conditions are favorable. The results highlight the effectiveness of integrating Sentinel-1 and Sentinel-2 data for classifying artificial forest types in complex tropical landscapes. The proposed methodology provides a transferable and scalable approach for monitoring other tropical plantation crops. Furthermore, the generated betel palm map offers valuable spatial information to support tropical agricultural structure optimization, land management, and policymaking.

## Figures and Tables

**Table 1:** Classification accuracy assessment based on validation samples.

	Total	Betel Palms	Non-Betel Palms	UA
Betel palms	1763	1532	231	86.89%
Non-betel palms	15,273	193	15,044	98.73%
PA		88.81%	98.49%	
OA = 97.51% kappa = 0.86				

**Table 2:** Detailed information on reference samples.

Land Cover and Land Use Types	Description	Number of ROIS	Detail Images in Google Earth
Betel palms	Lower canopy density than that of forests and rubber plantations. Some bare ground can be observed among betel palm rows.	766	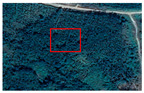
Forests	Closed canopy forest with continuous tree cover, with rare anthropogenic disturbance.	257	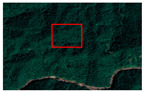
Rubber plantations	Closed and fairly closed canopy rubber plantations having obvious texture features.	771	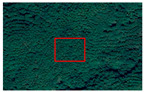
Croplands	Paddy fields or dry lands.	213	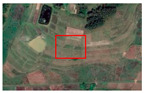
Built-up areas	Mainly include buildings, roads or residential regions.	113	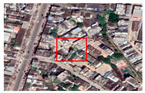
Water	Mainly include rivers, lakes or reservoirs.	105	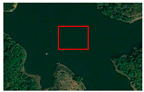

**Table 3:** Original features derived from the Sentinel-1 dataset.

Spectral Bands/ Vegetation Index (VI)	Abbreviation	Formula/Spatial Resolution
vertically polarized backscatter	*VV*	10 m
cross-polarized, vertical transmitter, horizontal receiver backscatter	*VH*	10 m
Average	AVE	(VV+VH)/2
Difference	DIF	VV−VH
Ratio1	RAT1	VV/VH
Ratio2	RAT2	VH/VV
Normalized Difference Index	NDI	$\frac{\left(VV-VH\right)}{\left(VV+VH\right)}$
NL Index	NL	$\frac{VV*VH}{\left(VV+VH\right)}$
GLCM Homogeneity	VV_Home, VH_Home	$\sum_{i,j=1}^{N}\frac{p_{i,j}}{1+{\left(i-j\right)}^{2}}$
GLCM Contrast	VV_Cont, VH_Cont	${\sum_{i,j=1}^{N}p_{i,j}\left(i-j\right)}^{2}$
GLCM Correlation	VV_Corr, VH_Corr	$\sum_{i,j=1}^{N}p_{i,j}\left(\frac{\left(i-\mu_{i}\right)\left(j-\mu_{j}\right)}{\sigma_{i}\sigma_{j}}\right)$
GLCM Mean	VV_Mean, VH_Mean	$\sum_{i,j=1}^{N}\frac{p_{i,j}}{N^{2}}$
GLCM Variance	VV_Vari, VH_Vari	${\sum_{i,j=1}^{N}p_{i,j}\left(i-\mu_{i}\right)}^{2}$
GLCM Dissimilarity	VV_Diss, VH_Diss	$\sum_{i,j=1}^{N}p_{i,j}\left|i-j\right|$
GLCM Entropy	VH_Entr, VV_Entr	$\sum_{i,j=1}^{N}p_{i,j}\left(-\ln p_{i,j}\right)$
GLCM Angular second moment	VH_Asm, VV_Asm	$\sum_{i,j=1}^{N}p_{i,j}^{2}$

Notes: *i*, *j* represent the row and column number of GLCM, respectively. *N* represents the whole number of rows or columns in the GLCM. *P_i_*,_*j*_ represents the normalized value of *i*, *j*. *p_i_*, *p_j_* means the marginal probability matrix with whole rows and columns of *P_i_*,*_j_*, respectively. *μ*_i_, *μ*_j_, *σ*_i_, and *σ*_i_ mean the averages and standard deviations of *p_i_*, *p_j_*.

**Table 4:** Original features derived from the Sentinel-2 dataset.

Spectral Bands/Vegetation Index (VI)	Abbreviation	Formula/Spatial Resolution
Blue	*B*_2_	10 m
Green	*B*_3_	10 m
Red	*B*_4_	10 m
Red Edge1	*B*_5_	20 m
Red Edge2	*B*_6_	20 m
Red Edge3	*B*_7_	20 m
Nir	*B*_8_	10 m
Red Edge4	*B*_8A_	20 m
SWIR1	*B*_11_	20 m
SWIR2	*B*_12_	20 m
Enhanced Vegetation Index	EVI	2.5 × (*B*_8_ − *B*_4_)/((*B*_8_ + 6 × *B*_4_ − 7.5 × *B*_2_) + 1)
Normalized Difference Vegetation Index	NDVI	(*B*_8_ − *B*_4_)/(*B*_8_ + *B*_4_)
Soil-Adjusted Total Vegetation Index	SATVI	$\frac{B_{11}-B_{4}}{B_{12}+B_{4}+0.1}\times \left(1.1-\frac{B_{12}}{2.0}\right)$
Red-edge Spectral Index	RSEI	(*B*_7_ + *B*_6_ − *B*_5_)/(*B*_5_ + *B*_6_ + *B*_7_)
Normalized Difference Tillage Index	NDTI	(*B*_11_ − *B*_12_)/(*B*_11_ + *B*_12_)
Normalized Difference Moisture Index	NDMI	(*B*_8_ − *B*_11_)/(*B*_8_ + *B*_11_)
Normalized Difference RE1	NDRE	(*B*_6_ − *B*_5_)/(*B*_6_ + *B*_5_)
Green Normalized Difference Vegetation Index	GNDVI	(*B*_8_ − *B*_3_)/(*B*_8_ + *B*_3_)
Green Chlorophyll Vegetation Index	GCVI	(*B*_8_/*B*_3_) − 1
Modified Crop Residue Cover	MODCRC	(*B*_11_ − *B*_3_)/(*B*_11_ + *B*_3_)
Simple Tillage Index	STI	*B*_11_/*B*_12_
Modified Normalized Difference Water Index	MNDWI	(*B*_3_ − *B*_11_)/(*B*_3_ + *B*_11_)
Normalized Difference NIR and SWIR1 Index	NDNS1	(*B*_8_ − *B*_11_)/(*B*_8_ + *B*_11_)
Normalized Difference NIR and SWIR2 Index	NDNS2	(*B*_8_ − *B*_12_)/(*B*_8_ + *B*_12_)
Greenness Index	GI	*B*_3_/*B*_4_
Green Vegetation Index	VIgreen	(*B*_3_ − *B*_4_)/(*B*_3_ + *B*_4_)
Simple Ratio Index	SRI	*B*_8_/*B*_4_
Modified Simple Ratio	MSR	$\frac{B_{8}/B_{4}-1}{{{(B}_{8}/B_{4})}^{0.5}+1}$
Modified Chlorophyll Absorption in Reflectance Index	MCARI	[(*B*_8_ − *B*_4_) − 0.2(*B*_8_ − *B*_3_)] × (*B*_8_/*B*_4_)
Triangular Vegetation Index	TVI	0.5 × [120 × (*B*_8_ − *B*_3_) − 200 × (*B*_4_ − *B*_3_)]
Transformed Chlorophyll Absorption in Reflectance Index	TCARI	3[(*B*_8_ − *B*_4_)−0.2 (*B*_8_ − *B*_3_) × (*B*_8_/*B*_4_)]
Medium-resolution Imaging Spectrometer Terrestrial Chlorophyll Index	MTCI	(*B*_6_ − *B*_5_)/(*B*_5_ − *B*_4_)
Chlorophyll Index Red Edge	CIRE	*B*_7_/*B*_5_ − 1
Chlorophyll Index Green	CIG	*B*_7_/*B*_3_ − 1

## Data Availability

The remote sensing datasets used in this study are publicly available via the Google Earth Engine platform, including Sentinel-1 Synthetic Aperture Radar data and Sentinel-2 multispectral imagery (https://earthengine.google.com). Sentinel-1 data source: https://developers.google.com/earth-engine/datasets/catalog/COPERNICUS_S1_GRD; Sentinel-2 data source: https://developers.google.com/earth-engine/datasets/catalog/COPERNICUS_S2_SR. The processed data supporting the findings of this study are available from the authors upon reasonable request.

## Abbreviations

The following abbreviations are used in this manuscript:

CLCD	China Land Cover Dataset
NGCC	National Geomatics Center of China
GEE	Google Earth Engine
LR	logistic regression
GNDVI	Green Normalized Difference Vegetation Index
GDP	Gross Domestic Product
SAR	Synthetic Aperture Radar
ML	Machine Learning
RF	Random Forest
SVM	Support Vector Machines
GBDT	Gradient Boosting Decision Trees
VH	vcross-polarized, vertical transmitter, horizontal receiver backscatter
VV	vertically polarized backscatter
GRD	Ground Range Data
AVE	Average
DIF	Difference
RAT1	Ratio1
RAT2	Ratio2
NDI	Normalized Difference Index
NL	NL Index
GLCM	Gray level Co-occurence Matrix
EVI	Enhanced Vegetation Index
NDVI	Normalized Difference Vegetation Index
SATVI	Soil-Adjusted Total Vegetation Index
RSEI	Red-edge Spectral Index
NDTI	Normalized Difference Tillage Index
NDMI	Normalized Difference Moisture Index
NDRE	Normalized Difference RE1
GCVI	Green Chlorophyll Vegetation Index
MODCRC	Modified Crop Residue Cover
STI	Simple Tillage Index
MNDWI	Modified normalized difference water index
NDNS1	Normalized Difference NIR and SWIR1 index
NDNS2	Normalized Difference NIR and SWIR2 index
GI	Greenness Index
VIgreen	Green Vegetation Index
SRI	Simple Ratio Index
MSR	Modified Simple Ratio
MCARI	Modified Chlorophyll Absorption in Reflectance Index
TVI	Triangular Vegetation Index
TCARI	Transformed Chlorophyll Absorption in Reflectance Index
MTCI	Medium resolution imaging spectrometer terrestrial chlorophyll index
CIRE	Chlorophyll Index Red Edge
CIG	Chlorophyll Index Green
OA	Overall Accuracy
UA	User’s Accuracy
PA	Producer’s Accuracy

## References

[1] Das M., Nath P.-C., Sileshi G.-W., Pandey R., Nath A.J., Das A.K. (2021). Biomass models for estimating carbon storage in Areca palm plantations. Environ. Sustain. Indic..

[2] Xu D., Lu Y.W., Liang H., Lu Z., Yu L.J., Liu Q. (2023). Areca Yellow Leaf Disease Severity Monitoring Using UAV-Based Multispectral and Thermal Infrared Imagery. Remote Sens..

[3] Li J., Cao X.-M., Jia X.-C., Liu L.-Y., Cao H.-W., Qin W.-Q., Li M. (2021). Iron Deficiency leads to chlorosis through impacting chlorophyll synthesis and nitrogen metabolism in *Areca catechu* L.. Front. Plant Sci..

[4] Li J., Jia X.-C., Liu L.-Y., Cao X.M., Xiong Y.-F., Yang Y.-D., Zhou H.-Q., Yi M., Li M. (2020). Comparative biochemical and transcriptome analysis provides insights into the regulatory mechanism of striped leaf albinism in arecanut (*Areca catechu* L.). Ind. Crops Prod..

[5] Sun R., Wu Z.X., Chen B.Q., Yang C., Qi D.-L., Lan G.-Y., Fraedrich K. (2020). Effects of land-use change on eco-environmental quality in Hainan Island. China. Ecol. Indic..

[6] Wu W.Y., Huang Z.-H., Sun Z.-Y., Zhang J., Wang S.-S., Fang M.-Y., Yang H., Lu H., Guo G.-L., Liu W.-J. (2024). Simulation and attribution analysis of terrestrial ecosystem carbon storage of Hainan Island from 2015 to 2050. Sci. Total Environ..

[7] Hernandez B.-Y., Zhu X.-M., Goodman M.-T., Gatewood R., Mendiola P., Quinata K., Paulino Y.-C. (2017). Betel nut chewing, oral premalignant lesions, and the oral microbiome. PLoS ONE.

[8] Luong J., Kozlakidis Z., Cheong I.-H., Wang H. (2023). Betel Nuts, health policies, and adolescent health. Innov. Digit. Health Diagn. Biomark..

[9] Chen S.-G., Dai S.-P., Hou Y.-Y. (2023). Learn from tobacco to reduce betel nut use. Science.

[10] Sidike P., Sagan V., Maimaitijiang M., Maimaitiyiming M., Shakoor N., Burken J., Mockler T., Fritschi F.B. (2019). dPEN: Deep progressively expanded network for mapping heterogeneous agricultural landscape using WorldView-3 satellite imagery. Remote Sens. Environ..

[11] Wang J., Xiao X.-M., Liu L., Wu X.-C., Qin Y.-W., Steiner J.-L., Dong J.-W. (2020). Mapping sugarcane plantation dynamics in Guangxi, China, by time series Sentinel-1, Sentinel-2 and Landsat images. Remote Sens. Environ..

[12] Li H.-Z., Zhao L.-L., Sun L.-Y., Li X.-L., Wang J., Han Y., Liang S.-Z., Chen J.-S. (2022). Capability of Phenology-Based Sentinel-2 Composites for Rubber Plantation Mapping in a Large Area with Complex Vegetation Landscapes. Remote Sens..

[13] Xia T., He Z., Cai Z.W., Wang C., Wang W.-J., Wang J.-Y., Hu Q., Song Q. (2022). Exploring the potential of Chinese GF-6 images for crop mapping in regions with complex agricultural landscapes. Int. J. Appl. Earth Obs. Geoinf..

[14] Zhu Z., Woodcock C.E. (2012). Object-based cloud and cloud shadow detection in Landsat imagery. Remote Sens. Environ..

[15] Hunt D.-A., Tabor K., Hewson J.-H., Wood M.-A., Reymondin L., Koening K., Harsh M.S., Follett F. (2020). Review of remote sensing methods to map coffee production systems. Remote Sens..

[16] Qadir A., Mondal P. (2020). Synergistic Use of Radar and Optical Satellite Data for Improved Monsoon Cropland Mapping in India. Remote Sens..

[17] Descals A., Szantol Z., Meijaard E., Sutikno H.S., Rindanata G., Wich S. (2019). Oil Palm (*Elaeis guineensis*) Mapping with Details: Smallholder versus Industrial Plantations and their Extent in Riau, Sumatra. Remote Sens..

[18] Maskell G., Chemura A., Nguyen H., Gornott C., Mondal P. (2021). Integration of Sentinel optical and radar data for mapping smallholder coffee production systems in Vietnam. Remote Sens. Environ..

[19] Peng Y.-F., Qiu B.-W., Tang Z.-H., Xu W.-M., Yang P., Wu W.-B., Chen X.-H., Zhu X.-L., Zhu P., Zhang X. (2024). Where is tea grown in the world: A robust mapping framework for agroforestry crop with knowledge graph and sentinels images. Remote Sens. Environ..

[20] Persson M., Lindberg E., Reese H. (2018). Tree Species Classification with Multi-Temporal Sentinel-2 Data. Remote Sens..

[21] Chen B.-Q., Li X.-P., Xiao X.-M., Zhao B., Dong J.-W., Kou W.-L., Qin Y.-W., Yang C., Wu Z.X., Sun R. (2016). Mapping tropical forests and deciduous rubber plantations in Hainan Island, China by integrating PALSAR 25-m and multi-temporal Landsat images. Int. J. Appl. Earth Obs. Geoinf..

[22] Sarzynski T., Giam X.-L., Carrasco L., Janice S. (2020). Combining radar and optical imagery to map oil palm plantations in Sumatra, Indonesia, using the google earth engine. Remote Sens..

[23] Deng X.-P., Guo S.-X., Sun L.-Y., Chen J.-S. (2020). Identification of short-rotation eucalyptus plantation at large Scale using multi-Satellite imageries and Cloud Computing Platform. Remote Sens..

[24] Nomura K., Mitchard E. (2018). More Than Meets the Eye: Using Sentinel-2 to Map Small Plantations in Complex Forest Landscapes. Remote Sens..

[25] Luo H.-X., Li M.-F., Dai S.-P., Li H.-L., Li Y.-P., Hu Y.-Y., Zheng Q., Yu X., Fang J.-H. (2022). Combinations of feature selection and Machine learning algorithms for object-oriented betel palms and mango plantations classification based on Gaofen-2 imagery. Remote Sens..

[26] Lebrini Y., Boudhar A., Hadria R., Lionboui H., Elmansouri L., Arrach R., Ceccato P., Benabdelouahab T. (2019). Identifying Agricultural Systems Using SVM Classification Approach Based on Phenological Metrics in a Semi-arid Region of Morocco. Earth Syst. Environ..

[27] Loggenberg K., Strever A., Greyling B., Poona N. (2018). Modelling Water Stress in a Shiraz Vineyard Using Hyperspectral Imaging and Machine Learning. Remote Sens..

[28] Arvor D., Betbeder J., Daher F., Blossier T., Roux R.-L., Corgne S., Corpetti T., Silgueiro V., Junior C. (2021). Towards user-adaptive remote sensing: Knowledge driven automatic classification of Sentinel-2 time series. Remote Sens. Environ..

[29] Zhao L.-C., Li Q.-Z., Chang Q.-R., Shang J.-L., Du X., Liu J.G., Dong T.F. (2022). In-season crop type identification using optimal feature knowledge graph. ISPRS J. Photogramm. Remote Sens..

[30] Teluguntla P., Thenkabail P.-S., Oliphant A., Xiong J., Gumma M.-K., Congalton R.-G., Yadav K., Huete A. (2018). A 30-m landsat-derived cropland extent product of Australia and China using random forest machine learning algorithm on Google Earth Engine cloud computing platform. ISPRS J. Photogramm. Remote Sens..

[31] Dong J.-W., Xiao X.-M., Menarguez M.-A., Zhang G.-L., Qin Y.-W., Thau D., Biradar C., Moore B. (2016). Mapping paddy rice planting area in northeastern Asia with Landsat 8 images, phenology-based algorithm and Google Earth Engine. Remote Sens. Environ..

[32] Xiong J., Thenkabail P.-S., Gumma M.-K., Teluguntla P., Poehnelt J., Congalton R.-G., Yadav K., Thau D. (2017). Automated cropland mapping of continental Africa using Google Earth Engine cloud computing. ISPRS J. Photogramm. Remote Sens..

[33] Zhou M., Han X., Wang J., Ji X., Zhou Y., Liu M. (2024). Identification and Mapping of Eucalyptus Plantations in Remote Sensing Data Using CCDC Algorithm and Random Forest. Forests.

[34] Tang C.X., Jiang X.D., Li G.Y., Lu D.S. (2024). Developing a New Method to Rapidly Map Eucalyptus Distribution in Subtropical Regions Using Sentinel-2 Imagery. Forests.

[35] HPBS (Hainan Provincial Bureau of Statistics), SONBSH (Survey Office of National Bureau of Statistics in Hainan) (2023). Hainan Statistical Yearbook 2023.

[36] Zhu Q.-H., Deng S.-W., Ma L., Li Q., Tan S.-H., Zheng Y.-J., Xu A.-Q., Wang H.-H. (2023). Research Progress on Processing Technology of Refined Betel Nut in China: A Review. Processes.

[37] Erijery J.-J., Singh M., Kent R. (2018). Mapping and assessment of vegetation types in the tropical rainforests of the Western Ghats using multispectral Sentinel-2 and SAR Sentinel-1 satellite imagery. Remote Sens. Environ..

[38] Cheng K., Su Y.-J., Guan H.-C., Tao S.-L., Ren Y., Hu T.-Y., Ma K.-P., Tang Y.-H., Guo Q.-H. (2023). Mapping China’s planted forests using high resolution imagery and massive amounts of crowdsourced samples. ISPRS J. Photogramm. Remote Sens..

[39] Gitelson A.-A., Kaufman Y.-J., Merzlyak M.-N. (1996). Use of a green channel in remote sensing of global vegetation from EOS-MODIS. Remote Sens. Environ..

[40] Yang J., Huang X. (2021). The 30m annual land cover dataset and its dynamics in China from 1990 to 2019. Earth Syst. Sci. Data.

[41] Chen J., Chen J., Liao A.-P., Cao X., Chen L.-J., Chen X.-H., He C.-Y., Han G., Peng S., Lu M. (2015). Global land cover mapping at 30 m resolution: A POK-based operational approach. ISPRS J. Photogramm. Remote Sens..

[42] Laliberte A.-S., Browning D.-M., Rango A. (2012). Comparison of three feature selection methods for object-based classification of sub-decimeter resolution UltraCam-L imagery. Int. J. Appl. Earth Obs. Geoinf..

[43] Ma L., Fu T.Y., Blaschke T., Li M.-C., Tiede D., Zhou Z.-J., Ma X.-X., Chen D.-L. (2017). Evaluation of feature selection methods for object-Based land cover mapping of unmanned aerial vehicle imagery using random forest and support vector machine classifiers. ISPRS Int. J. Geo-Inf..

[44] Dong J.-W., Xiao X.-M., Chen B.-Q., Tarbick N., Jin C., Zhang G.-L., Biradar C. (2013). Mapping deciduous rubber plantations through integration of PALSAR and multi-temporal Landsat imagery. Remote Sens. Environ..

[45] Gitelson A.-A., Vina A., Ciganda V., Rundquist D.-C., Arkebauer T.-J. (2005). Remote estimation of canopy chlorophyll content in crops. Geophys. Res. Lett..

[46] Wang X.-Q., Blanken P.-D., Wood J.-D., Nouvellon Y., Thaler P., Kasemsap P., Chidthaisong A., Petchprayoon P., Chayawat C., Xiao J.-F. (2023). Solar-induced chlorophyll fluorescence detects photosynthesis variations and drought effects in tropical rubber plantation and natural deciduous forests. Agric. For. Meteorol..

[47] Ferreira M.-P., Wagner F.-H., Aragao L.-E., Shimabukuro Y.-E., Filho C.-R. (2019). Tree species classification in tropical forests using visible to shortwave infrared WorldView-3 images and texture analysis. ISPRS J. Photogramm. Remote Sens..

[48] Ahuja S.-C., Ahuja U. (2012). Betel leaf and betel nut in Folklore on cultivation. Asian Agri-Hist..

[49] Cai Y.-P., Guan K.-Y., Peng J., Wang S.-W., Seifert C., Wardlow B., Li Z. (2018). A high-performance and in-season classification system of field-level crop types using time-series Landsat data and a machine learning approach. Remote Sens. Environ..

[50] You N.-S., Dong J.-W. (2020). Examining earliest identifiable timing of crops using all available Sentinel 1/2 imagery and Google Earth Engine. ISPRS J. Photogramm. Remote Sens..

[51] Xiao C.-W., Li P., Feng Z.-M., Liu Y.-Y., Zhang X.-Z. (2020). Sentinel-2 red-edge spectral indices (RESI) suitability for mapping rubber boom in Luang Namtha Province, northern Lao PDR. Int. J. Appl. Earth Obs. Geoinf..

[52] EI Hajj M.-M., Almashharawi S.-K., Johansen K., Elfarkh J., McCabe M.-F. (2022). Exploring the use of synthetic aperture radar data for irrigation management in super high-density olive orchards. Int. J. Appl. Earth Obs. Geoinf..

[53] Su W., Zhang C., Yang J.Y., Wu H.-G., Deng L., Ou W.-H., Yue A.-Z., Chen M.-J. (2012). Analysis of wavelet packet and statistical textures for object-oriented classification of forest agriculture ecotones using SPOT 5 imagery. Int. J. Remote Sens..

[54] Dai S.-P., Luo H.-X., Hu Y.-Y., Zheng Q., Li H.-L., Li M.-F., Yu X., Chen B.-Q. (2023). Retrieving leaf area index of rubber plantation in Hainan Island using empirical and neural network models with Landsat images. J. Appl. Remote Sens..

[55] Yan W.-H., Ouyang J.-J. (2011). Analysis on measurement and comparison of various vegetation canopy LAI in seacoast wetland, South China. Adv. Mater. Res..

[56] Tang H., Dubayah R., Swatantran A., Hofton M., Sheldon S., Clark D.-B., Blair B. (2012). Retrieval of vertical LAI profiles over tropical rain forests using waveform lidar at La Selva, Costa Rica. Remote Sens. Environ..

[57] Yang G.X., Li X.R., Liu P.Z., Yao X., Zhu Y., Cao W.X., Cheng T. (2023). Automated in-season mapping of winter wheat in China with training data generation and model transfer. ISPRS J. Photogramm. Remote Sens..

[58] Descals A., Wich S., Szantoi Z., Struebig M.J., Dennis R., Hatton Z., Ariffin T., Unus N., Gaveau D.L.A., Meijaard E. (2023). High-resolution global map of closed-canopy coconut palm. Earth Syst. Sci. Data.

[59] Chen B.Q., Xiao X.M., Wu Z.X., Yun T., Kou W.L., Ye H.C., Lin Q.H., Doughty R., Dong J.W., Ma J. (2018). Identifying Establishment Year and Pre-Conversion Land Cover of Rubber Plantations on Hainan Island, China Using Landsat Data during 1987–2015. Remote Sens..

[60] Ghorbanian A., Kakooei M., Amani M., Mahdavi S., Mohammadzadeh A., Hasanlou M. (2020). Improved land cover map of Iran using Sentinel imagery within Google Earth Engine and a novel automatic workflow for land cover classification using migrated training samples. ISPRS J. Photogramm. Remote Sens..

[61] DeAlban J.-D.T., Connette G.-M., Oswald P., Webb E.-L. (2018). Combined Landsat and L-Band SAR Data Improves Land Cover Classification and Change Detection in Dynamic Tropical Landscapes. Remote Sens..

[62] Li G.Y., Lu D.S., Moran E., Dutra L., Batistella M. (2012). A comparative analysis of ALOS PALSAR L-band and RADARSAT-2 C-band data for land-cover classification in a tropical moist region. ISPRS J. Photogramm. Remote Sens..

[63] Kakade A., Kumari B., Dholaniya P.-S. (2018). Feature selection using logistic regression in case-control DNA methylation data of Parkinson’s disease: A comparative study. J. Theor. Biol..

[64] FAO (2020). Global Forest Resources Assessment 2012.

[65] Dong J.-W., Xiao X.-M., Sheldon S., Biradar C., Duong N.D., Hazarika M. (2012). A comparison of forest cover maps in Mainland Southeast Asia from multiple sources: PALSAR, MERIS, MODIS and FRA. Remote Sens. Environ..

[66] Qin Y.-W., Xiao X.-M., Dong J.-W., Zhang G.-L., Shimada M., Liu J.-Y., Li C.-G., Kou W.-L., Moore B. (2015). Forest cover maps of China in 2010 from multiple approaches and data sources: PALSAR, Landsat, MODIS, FRA, and NFI. ISPRS J. Photogramm. Remote Sens..

[67] Ni W.J., Sun G.-Q., Guo Z.-F., Zhang Z.-Y., He Y.-T., Huang W.-L. (2013). Retrieval of forest biomass from ALOS PALSAR data using a lookup table method. IEEE J. Sel. Top. Appl. Earth Obs. Remote Sens..

[68] Zhang X.-M., He G.-J., Zhang Z.-M., Peng Y., Long T.-F. (2017). Spectral spatial multi-feature classification of remote sensing big data based on a random forest classifier for land cover mapping. Clust. Comput..

[69] Ghosh A., Joshi P.-K. (2014). Comparison of selected classification algorithms for mapping bamboo patches in lower Gangetic plains using very high resolution WorldView 2 imagery. Int. J. Appl. Earth Obs..

[70] Congalton R.G. (1991). A review of assessing the accuracy of classification of remotely sensed data. Remote Sens. Environ..

